# Empirical Antibiotic Therapy in Chronic Endometritis With and Without Focal Lesions: A Prospective Cohort Study

**DOI:** 10.3390/medsci13040278

**Published:** 2025-11-21

**Authors:** Iwona Gawron, Lucja Zaborowska, Kamil Derbisz, Inga Ludwin, Artur Ludwin

**Affiliations:** 1 Chair of Gynecology and Obstetrics, Faculty of Medicine, Jagiellonian University Medical College, Kopernika 23, 31-501 Krakow, Poland; 2 Clinical Department of Gynecological Endocrinology and Gynecology, University Hospital in Krakow, Kopernika 23, 31-501 Krakow, Poland; 3 1st Department of Obstetrics and Gynecology, Medical University of Warsaw, Starynkiewicza Sq 1/3, 02-015 Warsaw, Poland; 4 Doctoral School of Medical and Health Sciences, Jagiellonian University Medical College, sw. Lazarza 16, 31-530 Krakow, Poland; 5 Ludwin & Ludwin Private Medical Center, Rakowicka 17/7, 31-510 Krakow, Poland

**Keywords:** chronic endometritis, antibiotic therapy, plasma cell density, obstetric outcomes, office hysteroscopy

## Abstract

**Objective:** This study aimed to evaluate the efficacy of empirical antibiotic therapy in treating chronic endometritis (CE) associated with abnormal uterine bleeding (AUB), infertility, or intrauterine lesions. **Methods:** The prospective cohort study involved 102 women undergoing outpatient hysteroscopy (OH), with immunohistochemical diagnosis of CE based on plasma cell density (PCD). Seventy-six of these women received empirical antibiotic therapy (ofloxacin and metronidazole), while 26 did not. A follow-up OH was conducted in the third cycle following the initial procedure. **Results:** Hysteroscopic polypectomy significantly reduced PCD regardless of antibiotic use (*p* = 0.009). In cases without focal lesions but exhibiting CE features, antibiotic therapy notably decreased PCD (*p* = 0.018). The incidence of certain histopathological features of CE, such as stromal edema and stromal cell compaction, was significantly lower in women treated with antibiotics (*p* = 0.014). Among intrauterine pathologies, endometrial polyps (*p* = 0.009) and cesarean scar defects (*p* = 0.011) significantly increased the risk of CE. Only spindled transformation of stromal cells with edema correlated significantly with elevated PCD (*p* = 0.022). Antibiotic therapy did not improve obstetric outcomes. **Conclusions:** Polypectomy alone reduced PCD without antibiotics, while antibiotic treatment significantly decreased PCD and resolved CE features in cases without focal lesions. Therefore, antibiotics may be prioritized for cases without focal lesions, whereas surgical intervention may be sufficient for CE associated with eligible pathologies.

## 1. Introduction

Chronic endometritis (CE) is a presumed immune-mediated factor contributing to infertility, associated with recurrent pregnancy loss (RPL) [1] and recurrent implantation failure (RIF) [2], which has become the focus of extensive research in reproductive medicine in recent years [3,4].

Although this condition is typically attributed to a microbial etiology [5], the biological mechanisms underlying chronic inflammation in the endometrium remain insufficiently understood [6]. However, it has been demonstrated that the concomitant abnormal expression of adhesion molecules and chemokines facilitates the infiltration of B cells into endometrial glands and stroma. A subset of these B cells differentiates into stromal plasma cells, which subsequently produce excessive amounts of immunoglobulin G, primarily of the G2 subclass, potentially impeding the process of embryo implantation [7]. The diagnostics of CE continues to pose a challenge, both in terms of clinical symptoms and the clinical presentation of the uterine cavity as visualized through office hysteroscopy (OH) [8], as well as regarding reference detection methods [9]. Furthermore, the attempt to effectively treat this condition through empirical antibiotic therapy, along with the anticipated improvement in obstetric outcomes that it may facilitate, is burdened by an even greater complexity [10]. Identifying conditions associated with CE in which antibiotic administration could yield benefits in terms of symptom control or improvement in obstetric outcomes might contribute to the optimization of management strategies for abnormal uterine bleeding (AUB) or infertility.

The objective of the study was to evaluate the efficacy of empirical antibiotic therapy in the treatment of CE associated with AUB, infertility, or intrauterine lesions in women undergoing OH, with a specific emphasis on the resolution of focal intrauterine alterations, plasma cell infiltration, and histological abnormalities, and enhancements in clinical symptoms and obstetric outcomes. The secondary objectives included evaluating the correlation between clinical symptoms, ultrasound and hysteroscopic findings with plasma cell density (PCD), as well as assessing the correlation between PCD and the presence of other immune cells in the endometrial infiltration.

## 2. Materials and Methods

A prospective cohort study was conducted among women undergoing OH for the diagnostic evaluation of intrauterine pathologies, AUB, and idiopathic infertility during the years 2021 to 2022. The study was approved by the Bioethics Committee of Jagiellonian University (no. 1072.6120.322.2020; approval date: 25 November 2020) and registered in the Clinicaltrials.gov protocol registration system (no. NCT05946655; approval date: 29 June 2023). The research was conducted in compliance with the Helsinki Declaration, and all participants provided informed written consent to participate.

### 2.1. Criteria for Participation in the Study

The following inclusion criteria were employed: (i) age between 18 and 45 years, (ii) no prior diagnosis or treatment for suspected uterine cavity pathology, (iii) absence of active infections in the reproductive tract. The exclusion criteria were as follows: (i) pelvic surgery performed within the six months preceding the hysteroscopy, (ii) confirmed diagnosis of pelvic endometriosis, (iii) administration of antibiotics or probiotics within three months prior to the hysteroscopy.

### 2.2. Preoperative Assessment and Ultrasound Imaging of the Uterine Cavity

Women were qualified for OH following a thorough medical history taking, verification of vaginal biocenosis, cervical cytology, and negative cervical swab for Chlamydia, Mycoplasma, and Ureaplasma DNA. The physical examination included vaginal speculum inspection, bimanual palpation, and standard ultrasound of the reproductive organs using a Samsung WS80A with Elite (Samsung Electronics, Suwon, Republic of Korea). The abnormalities identifiable on ultrasound were classified as suspected intrauterine polyp, submucosal fibroid, polypoid endometrium and cesarean scar defect (CSD). The presence of an endometrial polyp was suspected when a hyperechoic focal mass was observed arising from the endometrial lining and extending into the endometrial cavity, with increased vascularity at the base, indicative of its blood supply. In contrast, a submucosal myoma appeared as a well-defined, heterogeneously echogenic mass reflecting their fibrous composition, located within the uterine wall, just beneath the endometrial lining, and extending into the endometrial cavity. The polypoid endometrium was visualized as isoechoic to hypoechoic in comparison to the normal endometrium, presenting a thickened endometrial lining with irregular or nodular contours, and lacking the well-defined mass characteristics typically associated with polyps. CSD was determined as a hypoechoic or anechoic irregular indentation in the anterior lower uterine segment, often accompanied by thinned and distorted residual myometrium, as well as fluid accumulation in the niche.

### 2.3. Conducting the Office Hysteroscopy

The procedure of OH was performed in the follicular phase of the menstrual cycle without general anesthesia through vaginoscopic approach. A Karl Storz Bettocchi rigid hysteroscope (Karl Storz SE & Co. KG, Tuttlingen, Germany) was utilized, featuring a 5 mm outer sheath, an operating channel compatible with 5 French instruments, and a 2.9 mm telescope equipped with a 30° angled lens. To ensure optimal visibility, uterine cavity was filled with a 0.9% NaCl solution through gravity inflow, supplemented by a manometer cuff inflated to a maximum pressure of 120 mmHg. Prior to the disruption of the endometrium, washings were collected from the uterine cavity for microbiological analysis, including aerobic and anaerobic cultures, as well as real-time polymerase chain reaction (RT-PCR) testing for the presence of Chlamydia, Mycoplasma, and Ureaplasma. The lavage samples were collected in Amies transport medium (Deltalab, Barcelona, Spain) for bacteriological examinations and in the Copan Universal Transport Medium (UTM-RT^®^) (Copan Diagnostics Inc., Murrieta, CA, USA) for RT-PCR analysis.

### 2.4. Hysteroscopic Detection of Intrauterine Pathologies

Pathologies detected during OH were classified into the following: polyps, polypoid endometrium, intrauterine adhesions, CSD, retained products of conception (RPOCs), submucosal fibroids, endometrial elevation and features of chronic endometritis, such as micropolyps, focal and diffuse hyperemia and endometrial edema. The polyp was diagnosed by visualizing a localized, rounded protrusion from the endometrial lining, while polypoid endometrium was recognized as thickened endometrial layer characterized by multiple broad-based projections resembling multiple polyps. Adhesions appeared as fibrous bands connecting different parts of the uterine lining, distorting the uterine cavity. CSD was identified as a wedge-shaped recess with thinned or absent endometrium in the area of a previous cesarean delivery. RPOCs were recognized based on the visualization of grayish-yellowish tissue fragments with a fleshy appearance. Fibroids were diagnosed after visualizing round and well-defined bulges with a smooth surface protruding into the uterine cavity. Endometrial elevation appeared as a local and smooth eminence of the endometrium. The hysteroscopic manifestations of CE were discerned using the Delphi criteria [11]. Following the inspection of the uterine cavity, tissue samples were collected for histopathological and immunohistochemical analysis. Focal lesions were excised employing either scissors or a loop or hook bipolar electrode. In the absence of focal lesions, random endometrial biopsies were performed using grasping forceps. The tissue material was pre-fixed in a 10% buffered formalin solution, then subjected to proper fixation and embedding in paraffin, and then cut and stained with eosin and hematoxylin for further detailed evaluation leading to the final diagnosis.

### 2.5. Histopathological Diagnostics

**Figure 1:**
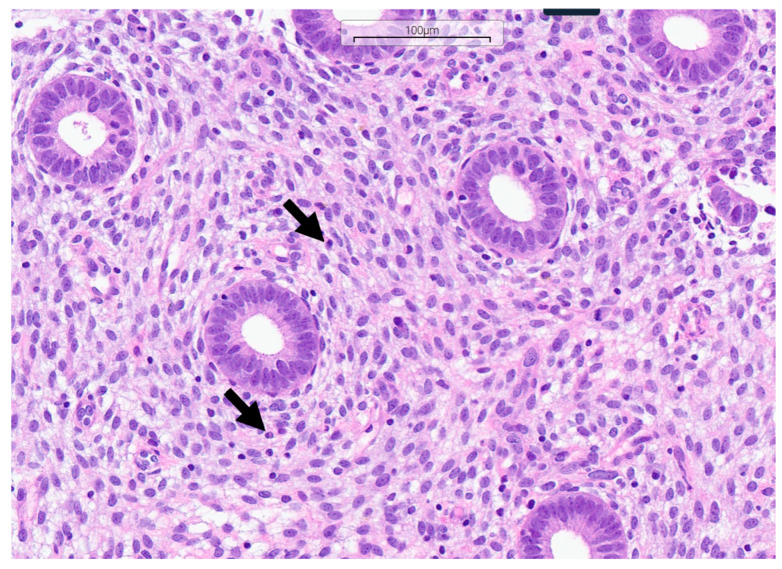
Formalin-fixed, paraffin-embedded tissue section of the endometrium stained with hematoxylin and eosin at 200-fold magnification. The plasma cells are indicated with arrows.

**Figure 2:**
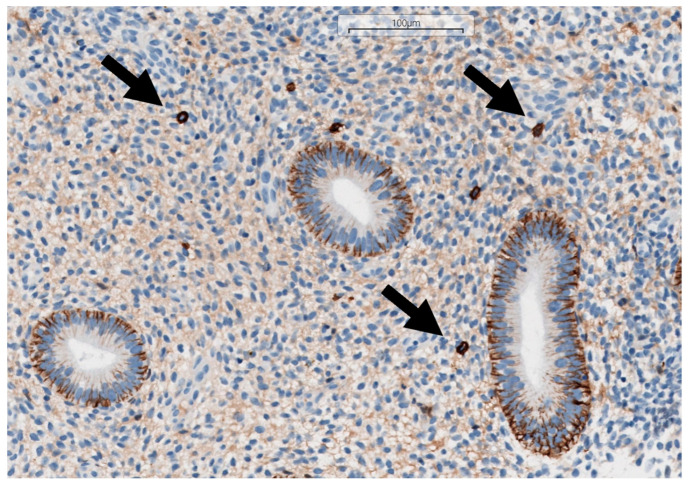
Immunohistochemically stained paraffin-embedded section of endometrial tissue, utilizing mouse monoclonal antibody CD138/syndecan-1 (B-A38) (Cell Marque-Sigma Aldrich, Merck KGaA, Darmstadt, Germany) and the ultraView DAB Detection Kit (Ventana Medical Systems, Inc., Tucson, AZ, USA), processed following the validated Roche protocol for the Benchmark Ultra slide staining system (F. Hoffmann-La Roche Ltd., Basel, Switzerland) at 200-fold magnification. The plasma cells are indicated with arrows.

The identification of CE was conducted using histopathological and immunohistochemical methodologies. Histopathological identification of CE relied on the detection of significant inflammatory infiltrate, alongside particular traits in the endometrial stroma and glands. Immunostaining was performed using the mouse monoclonal antibody CD138/syndecan-1 (B-A38) (Cell Marque-Sigma Aldrich, Merck KGaA, Darmstadt, Germany), a marker for plasma cells. Three-micrometer paraffin sections were stained using standardized automated techniques using the ultraView DAB Detection Kit (Ventana Medical Systems, Inc., Tucson, AZ, USA) according to Roche’s validated protocol on the Benchmark Ultra slide staining system (F. Hoffmann-La Roche Ltd., Basel, Switzerland). The identification of plasma cells using hematoxylin and eosin staining is illustrated in Figure 1, while the detection using immunohistochemical staining is depicted in Figure 2. The detection of at least one CD138-positive cell in a single high-power field (HPF) supported the immunohistochemical diagnosis of CE [12].

The pathologist remained blinded to the clinical data.

### 2.6. Empirical Antibiotic Therapy

Women with a positive immunohistochemical result who consented to antibiotic therapy received the treatment in the subsequent cycle. In weighing the absence of pathogen identification and the consequent necessity for the administration of empirical broad-spectrum antibiotic therapy with uncertain efficacy against the risks of adverse effects—including anaphylaxis, Clostridioides difficile infection, and the development of antibiotic resistance—the decision to proceed with the treatment arm was left to the women, who were provided with comprehensive counseling beforehand. The antibiotic therapy involved the oral administration of ofloxacin at a dosage of 200 mg twice daily and the vaginal administration of metronidazole at a daily dose of 500 mg, both for a duration of 10 days. A follow-up OH with repeated endometrial sampling was performed in the third cycle after the primary OH. Obstetric data and information on uterine bleeding were collected during a 24-month observation period following hysteroscopy.

### 2.7. Statistical Analysis

The analysis of quantitative variables was conducted through the calculation of descriptive statistics, including the mean, standard deviations, median, quartiles, and both minimum and maximum values. For qualitative variables, both absolute and percentage frequencies of all potential values were computed to facilitate a thorough analysis. The assessment of the normality of the distribution was performed using the Kolmogorov–Smirnov test. Comparative analyses of qualitative variable values across groups were carried out using the chi-square test (with Yates’ correction applied for 2 × 2 tables) or Fisher’s exact test when the assumptions related to expected frequencies for the chi-square test were not met. The comparison of quantitative variable values between two groups was implemented using the Mann–Whitney U test. In cases involving three or more groups, the Kruskal–Wallis test was employed, followed by Dunn’s post hoc test in the presence of statistically significant differences among groups. Spearman’s correlation coefficient was utilized to assess correlations among quantitative variables. The Wilcoxon signed-rank test for paired data was employed to compare the values of quantitative variables across two repeated measurements. In instances concerning qualitative variables evaluated through repeated measurements, McNemar’s test was applied, given that all analyzed variables were restricted to only two possible values. The cut-off point for evaluating the influence of a quantitative variable on a dichotomous variable was established based on the distance from the upper left corner of the ROC curve. A significance level of 0.05 was predetermined for this analysis, indicating that all *p*-values below this threshold were interpreted as indicative of statistically significant relationships. The analysis was performed using R software, version 4.4.2 [13].

## 3. Results

**Figure 3:**
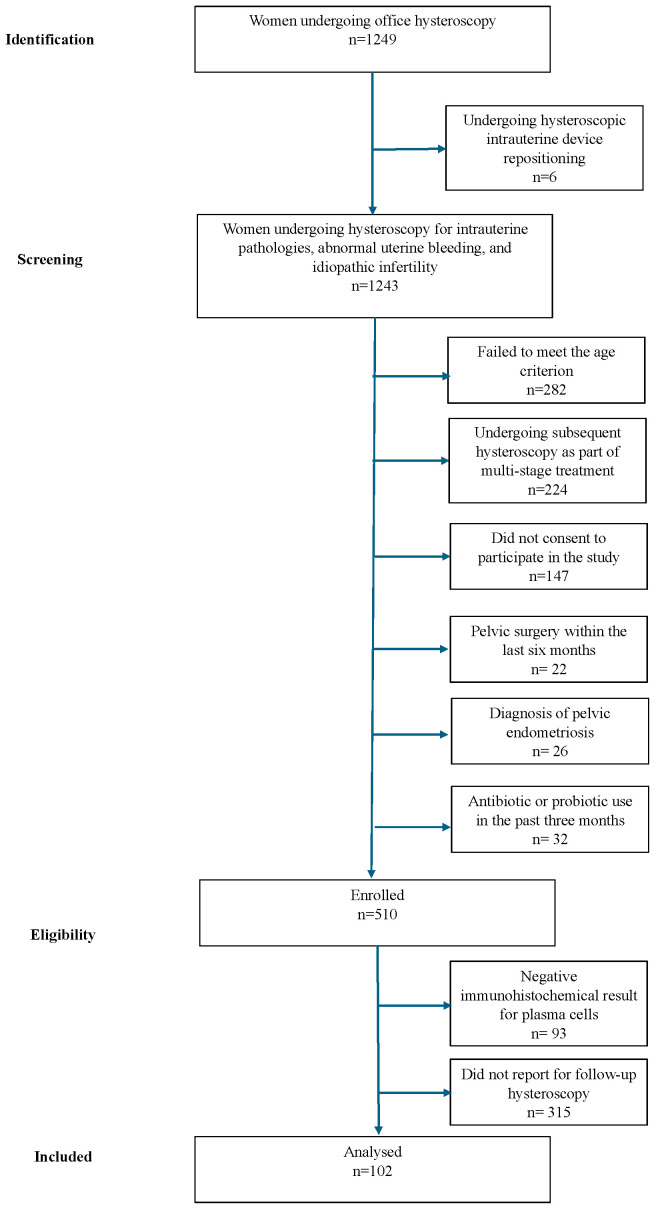
Flow diagram illustrating the recruitment process for the study.

The recruitment of participants, who were included consecutively, is presented in the flow diagram (Figure 3). The study group involved 102 women, of whom 76 underwent empiric antibiotic therapy following OH, while 26 did not receive antibiotics. The collected database was made publicly available in Harvard Dataverse at https://doi.org/10.7910/DVN/B0BGKU, accessed on 15 April 2025.

The comparative characteristics of both arms of the study are delineated in Table 1.

The rates of primary and secondary infertility were higher in women who received antibiotics (*p* < 0.001). Miscarriage rates were also significantly greater among women who received antibiotics (*p* = 0.005). Conversely, the percentage of polyps detected via ultrasound (*p* < 0.001) and primary OH (*p* = 0.002) was higher in women who did not receive antibiotics. Additionally, the incidence of endometrial stromal edema and stromal cell compaction in tissue specimens from primary OH was greater in women who received antibiotics (*p* = 0.037).

The frequency of specific intrauterine lesions identified during follow-up OH—which exhibited persistence by reappearing following their removal—in women not treated with antibiotics, in those who received antibiotic therapy, and irrespective of antibiotic treatment is delineated in Table 2.

In the analysis of women who did not receive antibiotics, all 7 women without polyps in the primary OH continued to have no lesions in the secondary OH (100.00%). Among the 19 women with polyps in the primary OH, 2 (10.53%) still had polyps in the secondary OH, while 17 (89.47%) did not. These changes were statistically significant (*p* < 0.001).

Among the women who received antibiotics, of the 46 individuals without polyps in the primary OH, 44 (95.65%) remained without such lesions in the secondary OH, while 2 (4.35%) exhibited polyps. Among the 27 women with polyps in the primary OH, 6 (22.22%) retained polyps in the secondary examination, whereas 21 (77.78%) did not. These changes showed statistical significance (*p* < 0.001).

Among the women not receiving antibiotics, 54 of the 58 individuals (93.10%) without diffuse hyperemia in the primary OH continued to show no abnormalities in the secondary OH, while 4 (6.90%) exhibited such findings. Among the 15 women with diffuse hyperemia in the primary OH, 1 (6.67%) still had this condition in the secondary OH, whereas 14 (93.33%) did not. These findings were statistically significant (*p* = 0.034).

Analyzing the entire cohort without considering antibiotic administration, of the 53 women without polyps in the primary OH, 51 (96.23%) continued to show no lesions in the secondary OH, while 2 (3.77%) exhibited polyps. Among the 47 women with polyps in the primary OH, 9 (19.15%) retained these lesions in the secondary OH, whereas 38 (80.85%) did not. These results were statistically significant (*p* < 0.001).

In the entire cohort, without considering the administration of antibiotic therapy, of the 81 women without diffuse hyperemia in the primary OH, 76 (93.83%) continued to show no such condition in the secondary OH, while 5 (6.17%) exhibited diffuse hyperemia. Among the 19 women with diffuse hyperemia in the primary OH, 1 (5.26%) still had this condition in the secondary OH, while 18 (94.74%) did not. These findings were statistically significant (*p* < 0.05).

For the other hysteroscopic findings, no significant associations were found concerning the persistence or occurrence of lesions based on the administration or non-administration of antibiotic therapy (all *p* > 0.05).

The comparison of PCD in tissue samples obtained during primary and follow-up OH across study arms, with respect to initially detected intrauterine lesions, is detailed in Table 3.

In cases of isolated polyp excision during the primary OH not followed by antibiotic therapy, PCD was significantly greater in tissue samples from the primary OH than in those from the secondary OH (*p* = 0.023).

In cases of isolated polyp excision during the primary OH, regardless of antibiotic use, PCD was significantly greater in tissue samples from the primary OH than in those from the secondary OH (*p* = 0.009).

In cases where the polyp excised during the primary OH was accompanied by hysteroscopic features of CE and without CSD, PCD in tissue samples from the primary OH was greater than that in samples from the secondary OH after subsequent antibiotic therapy (*p* = 0.033).

A similar decrease in PCD in samples from the secondary OH was observed in cases with isolated hysteroscopic features of CE in the primary OH following subsequent antibiotic therapy (*p* = 0.018) and regardless of antibiotic use (*p* = 0.009), resulting from the significant number of participants receiving antibiotics.

No significant associations were found for the other combinations of uterine lesions (all *p* > 0.05).

The comparison of PCD in tissue samples obtained during primary and follow-up OH across study arms, in relation to the pattern of AUB, is presented in Table 4.

In women who did not receive antibiotics, PCD did not differ significantly between tissue samples from primary and secondary OHs in cases without AUB, in cases with HMB, IMB, or in cases with both HMB and IMB (all *p* > 0.05). Considering the entire cohort of women not receiving antibiotics, PCD was significantly greater in tissue samples from the primary compared to the secondary OH (*p* = 0.031), regardless of AUB.

Among women receiving antibiotics, PCD was significantly greater in tissue samples obtained during the primary hysteroscopy compared to the secondary hysteroscopy in cases without AUB (*p* = 0.006), in cases with HMB (*p* = 0.012), and in the entire group regardless of AUB (*p* < 0.001).

Similar associations were observed when analyzing the entire cohort regardless of antibiotic use (*p* < 0.001, *p* = 0.033, and *p* < 0.001, respectively).

The persistence of specific histopathological features in the endometrium in primary and follow-up OH across study arms is outlined in Table 5.

The frequency of specific histological changes in tissue samples obtained during the primary and secondary OHs did not differ significantly in the group of women who did not receive antibiotics (all *p* > 0.05).

Considering all women who received antibiotics, of the 59 individuals without stromal edema and stromal cell compaction in the tissue biopsy from the primary OH, 55 (93.22%) remained free of these features in the secondary OH, while 4 (6.78%) exhibited them. Among the 17 women with these features in the primary OH, 1 (5.88%) still had them in the secondary examination, while 16 (94.12%) did not. These changes were statistically significant (*p* = 0.014). Among the 67 women without isolated edema in the tissue sample from the primary OH, 48 (71.64%) remained free of this histological feature in the secondary OH, while 19 (28.36%) exhibited it. Of the 9 women with this feature in the primary OH, 3 (33.33%) still showed it in the secondary OH, while 6 (66.67%) did not. These findings were statistically significant (*p* = 0.016). Similar patterns in the occurrence of stromal edema and stromal cell compaction (*p* = 0.012) and isolated edema (*p* = 0.045) were observed when analyzing the entire cohort, regardless of antibiotic use.

The analyses of the associations between clinical symptoms, ultrasound findings, hysteroscopically identified lesions, and PCD are depicted in Table 6.

PCD in tissue samples from the primary OH was significantly greater in women with ultrasound-detected CSD compared to those without (*p* = 0.019), as well as in those with hysteroscopically confirmed CSD (*p* = 0.011). Additionally, PCD was significantly higher in women with hysteroscopically visible polyps than in those without (*p* = 0.009). The remaining variables did not demonstrate a significant correlation with PCD (all *p* > 0.05).

Additionally, no significant correlations were found between quantitative variables such as the number of pregnancies, deliveries or miscarriages (Spearman’s correlation coefficient: r = −0.146, *p* = 0.141; r = −0.072, *p* = 0.47; r = −0.113, *p* = 0.255, respectively) and PCD.

The analysis of associations between PCD and the presence of other immune cells within the endometrial infiltration, along with the histopathological characteristics of the endometrium, is detailed in Table 7.

PCD in endometrial biopsies from the primary OH was significantly greater in women with spindled transformation and/or elongation of stromal cells and edema compared to those without this histological feature (*p* = 0.022). The remaining histological features did not show a significant correlation with PCD (all *p* > 0.05).

The cutoff for PCD indicative of CE in histopathology was calculated. The area under the receiver operating characteristic curve (ROC) equaled to 0.484 (AUC), indicating that PCD was a poor predictor of histopathological features of CE. The optimal cutoff for PCD was established at 6/1 HPF, with the criterion indicating that a cell count exceeding this threshold suggested a likelihood of CE with a sensitivity of 49.15% and a specificity of 50.00%. No lavage sample was positive for anaerobic pathogens or atypical bacteria. However, samples for aerobic pathogens tested positive in 31 women. The cultured species and the number of cases were as follows: *Coagulase-Negative Staphylococcus* (12), *Lactobacillus* sp. (5), *Escherichia coli* (2), *Staphylococcus hominis* (1), *Staphylococcus saprophyticus* (1), *Staphylococcus aureus* (1), *Streptococcus agalactiae* (1), *Streptococcus gallolyticus* (1), *Klebsiella Oxytoca* (1), *Lactobacillus reuteri* (1), *Lactobacillus acidophilus* (1), *Lactobacillus casei* (1), *Lactobacillus paraplantarum* (1), *Actinotignum schaalii* (1), and *Leuconostoc citreum* (1). Due to the limited prevalence of specific microbiological results, statistical analysis was not feasible.

The treatment outcomes in terms of obstetric and symptomatic aspects are presented in Table 8.

No significant differences were found in obstetric outcomes following the secondary hysteroscopy based on the administration of antibiotics, regardless of infertility type or IVF treatment (all *p* > 0.05). Additionally, no significant differences were observed in the persistence of AUB following the secondary hysteroscopy based on antibiotic use (all *p* > 0.05).

No cases of uterine perforation, allergy to the administered antibiotics, or uterine malignancy were identified.

## 4. Discussion

The regression of CE following polypectomy aligned with research indicating that CE associated with intrauterine lesions resolved post-removal without antibiotics. This was particularly evident in polypectomy cases, where obstetric outcomes also improved without subsequent antibiotic therapy [14]. Conversely, certain studies did not demonstrate significant differences in spontaneous pregnancy rates or time to conception between women with CE and those without CE following reproductive surgery [15]. Additionally, some research indicated that CE persisted despite antibiotic treatment [16] and that such treatment did not enhance obstetric outcomes [17,18]. Other reports emphasized that antibiotic therapy may improve IVF outcomes only when the resolution of CE was biopsy-confirmed [19], while another study indicated that CE did not influence IVF outcomes in cases of RIF [1]. No consensus has yet been reached on the effectiveness of empiric antibiotic therapy in the treatment of CE in the context of improving medically assisted reproduction outcomes, as several studies have failed to substantiate significant benefits [20,21,22]. Indeed, in this study the administration of antibiotics in the postoperative period did not exert any statistically significant influence on obstetric outcomes, irrespective of whether the case involved primary or secondary infertility, nor did it impact the results of IVF. On the other hand, certain reports indicated that the implementation of empirical antibiotic therapy resulted in the resolution of histopathological manifestations of CE and even contributed to an improvement in obstetric outcomes in terms of spontaneous conceptions [23]. Indeed, addressing CE with antibiotics in the absence of focal lesions has led to the regression of histopathological characteristics of CE and a significant reduction in PCD. Nevertheless, this did not lead to a substantial improvement in obstetric outcomes.

In the analysis of the correlation between selected variables and PCD, only endometrial polyps, CSD and spindled transformation and/or elongation of stromal cells with edema significantly increased the risk of CE, underscoring the previously declared limited diagnostic concordance among various methods of CE detection [9]. Similarly, microbiological techniques did not demonstrate adequate sensitivity and specificity [9], as the cultured pathogens were primarily attributed to sample contamination by incidental flora.

Empirical antibiotic therapy may provide benefits in specific cases of idiopathic infertility or HMB but also poses risks of adverse effects if misused. Polypectomy effectively reduces PCD without antibiotics, while antibiotic therapy significantly decreases PCD and addresses histopathological features of CE in cases without focal lesions. However, antibiotics do not enhance obstetric outcomes. Endometrial polyps and CSD significantly increase the risk of CE, but unlike polypectomy, hysteroscopic correction of CSD does not reduce PCD. It is apparent that hysteroscopic resection of the protruding tissue at the edge of the minor CSD, along with coagulation of the niche via OH, fails to achieve complete ablation of the altered tissue, resulting in ongoing inflammation that remains unresponsive to antibiotic therapy. Hysteroscopic visual assessment of the uterine cavity should not replace immunohistochemistry in diagnosing CE.

The health effects of CE in the field of reproductive health have recently garnered considerable research attention, with a focus on identifying specific symptoms and risk factors [24], advancing diagnostic methodologies, developing therapeutic strategies, and mitigating alleged adverse reproductive outcomes. Although various gynecological pathologies associated with CE have been identified, including endometrial polyps, intrauterine adhesions, CSD, tubal obstruction and endometriosis [25], the extensive body of research has yet to establish a consensus regarding diagnostic criteria [12,26], the significance of CE in relation to obstetric outcomes [1,21,27,28], and standardization of therapeutic practices [17].

Since polypectomy alone proved to be an adequate therapeutic intervention in cases of concurrent CE, it can be inferred that CE associated with idiopathic infertility or recurrent obstetric failures may have a different etiology than that related to intrauterine lesions such as polyps, warranting further investigation. It remains uncertain whether empirical antibiotic therapy resolves CE or improves clinical outcomes, and whether antibiotics are warranted after hysteroscopic resection of CE-related intrauterine pathologies or in cases without focal lesions identified by endometrial biopsy. Future randomized studies, followed by meta-analyses, may provide clarification on the optimized therapeutic approaches for CE associated with various gynecological pathologies.

The interpretation of research outcomes in the explored area is hampered by inconsistencies in CE definitions, small cohorts, varying antibiotic regimens, and the inclusion of additional endometrial conditioning procedures [15]. The strength of this study lies in its prospective design, comprehensive data collection, and multifaceted examination of parameters. Notably, the immunohistochemical diagnosis cutoff for CE was set at one plasma cell per HPF, rather than 5/10 HPF or 1/10 HPF, as reported in various publications [12], reflecting the research center’s experience [29]. This approach likely reduced false-positive results, considering that plasma cells are also present in healthy endometrium [30]. However, like most research in this field, this study faced limitations due to a small sample size and the lack of randomization imposed by hospital policies. The voluntary nature of participation and assignment to a specific arm of the study, alongside the predominance of women undergoing antibiotic therapy—who reported infertility more frequently and were less often diagnosed with endometrial polyps—may have influenced the results as a consequence of the study’s non-randomized design. To address these limitations, a future randomized controlled trial should be designed, incorporating meticulous selection of both the study population and the control group.

## Figures and Tables

**Table 1:** The comparative characteristics of the population with respect to both arms of the study, based on medical history data, ultrasonographic assessments, hysteroscopic evaluations and histopathological examinations.

Variable		Antibiotic Therapy			*p*
		No (N = 26)	Yes (N = 76)	Total (N = 102)	
Age [years]	Mean (SD)	33.96 (5.9)	35.13 (4.46)	34.83 (4.86)	*p* = 0.372
	Median (quartiles)	34.5 (31–36)	35 (32–38)	35 (32–38)	
	Range	22–44	24–45	22–45	
	n	26	76	102	
Abnormal uterine bleeding	No	8 (30.77%)	39 (51.32%)	47 (46.08%)	*p* = 0.113
	Yes	18 (69.23%)	37 (48.68%)	55 (53.92%)	
Heavy menstrual bleeding	No	11 (42.31%)	50 (65.79%)	61 (59.80%)	*p* = 0.061
	Yes	15 (57.69%)	26 (34.21%)	41 (40.20%)	
Intermenstrual bleeding	No	19 (73.08%)	59 (77.63%)	78 (76.47%)	*p* = 0.838
	Yes	7 (26.92%)	17 (22.37%)	24 (23.53%)	
Dysmenorrhea	No	21 (80.77%)	58 (76.32%)	79 (77.45%)	*p* = 0.844
	Yes	5 (19.23%)	18 (23.68%)	23 (22.55%)	
Pelvic pain syndrome	No	25 (96.15%)	75 (98.68%)	100 (98.04%)	*p* = 0.447
	Yes	1 (3.85%)	1 (1.32%)	2 (1.96%)	
Infertility	No	17 (65.38%)	11 (14.47%)	28 (27.45%)	*p* < 0.001 *
	Primary	5 (19.23%)	22 (28.95%)	27 (26.47%)	
	Secondary	4 (15.38%)	43 (56.58%)	47 (46.08%)	
Pregnancies	Mean (SD)	1.04 (1.11)	1.2 (1.33)	1.16 (1.27)	*p* = 0.729
	Median (quartiles)	1 (0–2)	1 (0–2)	1 (0–2)	
	Range	0–3	0–5	0–5	
	n	26	76	102	
Childbirths	Mean (SD)	0.81 (1.02)	0.42 (0.66)	0.52 (0.78)	*p* = 0.1
	Median (quartiles)	0 (0–1.75)	0 (0–1)	0 (0–1)	
	Range	0–3	0–2	0–3	
	n	26	76	102	
Miscarriages	Mean (SD)	0.23 (0.65)	0.78 (1.09)	0.64 (1.02)	*p* = 0.005 *
	Median (quartiles)	0 (0–0)	0 (0–1)	0 (0–1)	
	Range	0–2	0–5	0–5	
	n	26	76	102	
US Polyp	No	6 (23.08%)	50 (65.79%)	56 (54.90%)	*p* < 0.001 *
	Yes	20 (76.92%)	26 (34.21%)	46 (45.10%)	
US Polypoid endometrium	No	23 (88.46%)	70 (92.11%)	93 (91.18%)	*p* = 0.69
	Yes	3 (11.54%)	6 (7.89%)	9 (8.82%)	
US Submucosal myoma	No	25 (96.15%)	75 (98.68%)	100 (98.04%)	*p* = 0.447
	Yes	1 (3.85%)	1 (1.32%)	2 (1.96%)	
US Cesarean scar defect	No	23 (88.46%)	68 (89.47%)	91 (89.22%)	*p* = 1
	Yes	3 (11.54%)	8 (10.53%)	11 (10.78%)	
H1 Polyp	No	7 (26.92%)	49 (64.47%)	56 (54.90%)	*p* = 0.002 *
	Yes	19 (73.08%)	27 (35.53%)	46 (45.10%)	
H1 Polypoid endometrium	No	18 (69.23%)	45 (59.21%)	63 (61.76%)	*p* = 0.5
	Yes	8 (30.77%)	31 (40.79%)	39 (38.24%)	
H1 Micropolyps	No	24 (92.31%)	57 (75.00%)	81 (79.41%)	*p* = 0.109
	Yes	2 (7.69%)	19 (25.00%)	21 (20.59%)	
H1 Endometrial edema	No	26 (100.00%)	75 (98.68%)	101 (99.02%)	*p* = 1
	Yes	0 (0.00%)	1 (1.32%)	1 (0.98%)	
H1 Focal hyperemia	No	20 (76.92%)	51 (67.11%)	71 (69.61%)	*p* = 0.489
	Yes	6 (23.08%)	25 (32.89%)	31 (30.39%)	
H1 Diffuse hyperemia	No	22 (84.62%)	60 (78.95%)	82 (80.39%)	*p* = 0.732
	Yes	4 (15.38%)	16 (21.05%)	20 (19.61%)	
H1 Retained products of conception	No	26 (100.00%)	75 (98.68%)	101 (99.02%)	*p* = 1
	Yes	0 (0.00%)	1 (1.32%)	1 (0.98%)	
H1 Intrauterine adhesions	No	25 (96.15%)	72 (94.74%)	97 (95.10%)	*p* = 1
	Yes	1 (3.85%)	4 (5.26%)	5 (4.90%)	
H1 Cesarean scar defect	No	22 (84.62%)	67 (88.16%)	89 (87.25%)	*p* = 0.735
	Yes	4 (15.38%)	9 (11.84%)	13 (12.75%)	
H1 Submucosal adenomyosis	No	26 (100.00%)	69 (90.79%)	95 (93.14%)	*p* = 0.186
	Yes	0 (0.00%)	7 (9.21%)	7 (6.86%)	
H1 Endometrial elevation	No	24 (92.31%)	72 (94.74%)	96 (94.12%)	*p* = 0.643
	Yes	2 (7.69%)	4 (5.26%)	6 (5.88%)	
	Yes	11 (42.31%)	20 (26.32%)	31 (30.39%)	
H1 Plasmocytes [n/1HPF]	Mean (SD)	12.35 (18.12)	11.83 (19.35)	11.96 (18.95)	*p* = 0.377
	Median (quartiles)	8 (4–12.75)	5 (3–10.25)	5.5 (3–11)	
	Range	1–94	1–126	1–126	
	n	26	76	102	
H1 Lymphocytic infiltration	No	17 (65.38%)	49 (64.47%)	66 (64.71%)	*p* = 1
	Yes	9 (34.62%)	27 (35.53%)	36 (35.29%)	
H1 Macrophages	No	22 (84.62%)	56 (73.68%)	78 (76.47%)	*p* = 0.386
	Yes	4 (15.38%)	20 (26.32%)	24 (23.53%)	
H1 Granulocytes	No	24 (92.31%)	75 (98.68%)	99 (97.06%)	*p* = 0.159
	Yes	2 (7.69%)	1 (1.32%)	3 (2.94%)	
H1 Spindled transformation and/or elongation of stromal cells and edema	No	21 (80.77%)	59 (77.63%)	80 (78.43%)	*p* = 0.952
	Yes	5 (19.23%)	17 (22.37%)	22 (21.57%)	
H1 Stromal edema and stromal cell compaction	No	25 (96.15%)	59 (77.63%)	84 (82.35%)	*p* = 0.037 *
	Yes	1 (3.85%)	17 (22.37%)	18 (17.65%)	
H1 Edema only	No	23 (88.46%)	67 (88.16%)	90 (88.24%)	*p* = 1
	Yes	3 (11.54%)	9 (11.84%)	12 (11.76%)	

*p*—Qualitative variables: chi-squared or Fisher’s exact test; Quantitative variables: Mann–Whitney test, * statistically significant (*p* < 0.05), US = identified through ultrasound, H1 = identified through primary office hysteroscopy, HPF = high-power field.

**Table 2:** The persistence of specific intrauterine lesions observed in primary and follow-up office hysteroscopy among women not treated with antibiotics, among those treated with antibiotics, and irrespective of antibiotic therapy.

Hysteroscopic Diagnosis	No Antibiotic Therapy				Antibiotic Therapy				Total			
Polyp		H1: No (N = 7)	H1: Yes (N = 19)			H1: No (N = 46)	H1: Yes (N = 27)			H1: No (N = 53)	H1: Yes (N = 47)	
	H2: No	7 (100.00%)	17 (89.47%)	*p* < 0.001 *	H2: No	44 (95.65%)	21 (77.78%)	*p* < 0.001 *	H2: No	51 (96.23%)	38 (80.85%)	*p* < 0.001 *
	H2: Yes	0 (0.00%)	2 (10.53%)		H2: Yes	2 (4.35%)	6 (22.22%)		H2: Yes	2 (3.77%)	9 (19.15%)	
Polypoid endometrium		H1: No (N = 18)	H1: Yes (N = 8)			H1: No (N = 44)	H1: Yes (N = 29)			H1: No (N = 62)	H1: Yes (N = 38)	
	H2: No	9 (50.00%)	5 (62.50%)	*p* = 0.423	H2: No	33 (75.00%)	16 (55.17%)	*p* = 0.441	H2: No	42 (67.74%)	21 (55.26%)	*p* = 1
	H2: Yes	9 (50.00%)	3 (37.50%)		H2: Yes	11 (25.00%)	13 (44.83%)		H2: Yes	20 (32.26%)	17 (44.74%)	
Micropolyps		H1: No (N = 24)	H1: Yes (N = 2)			H1: No (N = 57)	H1: Yes (N = 19)			H1: No (N = 81)	H1: Yes (N = 22)	
	H2: No	18 (75.00%)	1 (50.00%)	*p* = 0.131	H2: No	49 (85.96%)	12 (63.16%)	*p* = 0.502	H2: No	67 (82.72%)	13 (59.09%)	*p* = 1
	H2: Yes	6 (25.00%)	1 (50.00%)		H2: Yes	8 (14.04%)	7 (36.84%)		H2: Yes	14 (17.28%)	9 (40.91%)	
Focal hyperemia		H1: No (N = 20)	H1: Yes (N = 6)			H1: No (N = 50)	H1: Yes (N = 23)			H1: No (N = 71)	H1: Yes (N = 29)	
	H2: No	13 (65.00%)	5 (83.33%)	*p* = 0.773	H2: No	38 (76.00%)	18 (78.26%)	*p* = 0.361	H2: No	52 (73.24%)	23 (79.31%)	*p* = 0.643
	H2: Yes	7 (35.00%)	1 (16.67%)		H2: Yes	12 (24.00%)	5 (21.74%)		H2: Yes	19 (26.76%)	6 (20.69%)	
Diffuse hyperemia		H1: No (N = 22)	H1: Yes (N = 4)			H1: No (N = 58)	H1: Yes (N = 15)			H1: No (N = 81)	H1: Yes (N = 19)	
	H2: No	22 (100.00%)	4 (100.00%)	*p* = 0.134	H2: No	54 (93.10%)	14 (93.33%)	*p* = 0.034 *	H2: No	76 (93.83%)	18 (94.74%)	*p* = 0.012 *
	H2: Yes	0 (0.00%)	0 (0.00%)		H2: Yes	4 (6.90%)	1 (6.67%)		H2: Yes	5 (6.17%)	1 (5.26%)	
Intrauterine adhesions		H1: No (N = 25)	H1: Yes (N = 1)			H1: No (N = 69)	H1: Yes (N = 4)			H1: No (N = 95)	H1: Yes (N = 5)	
	H2: No	25 (100.00%)	1 (100.00%)	*p* = 1	H2: No	69 (100.00%)	3 (75.00%)	*p* = 0.248	H2: No	95 (100.00%)	4 (80.00%)	*p* = 0.134
	H2: Yes	0 (0.00%)	0 (0.00%)		H2: Yes	0 (0.00%)	1 (25.00%)		H2: Yes	0 (0.00%)	1 (20.00%)	
Endometrial elevation		H1: No (N = 24)	H1: Yes (N = 2)			H1: No (N = 69)	H1: Yes (N = 4)			H1: No (N = 94)	H1: Yes (N = 6)	
	H2: No	23 (95.83%)	2 (100.00%)	*p* = 1	H2: No	64 (92.75%)	4 (100.00%)	*p* = 1	H2: No	88 (93.62%)	6 (100.00%)	*p* = 1
	H2: Yes	1 (4.17%)	0 (0.00%)		H2: Yes	5 (7.25%)	0 (0.00%)		H2: Yes	6 (6.38%)	0 (0.00%)	
Endometrial edema	Not applicable ^a^					H1: No (N = 72)	H1: Yes (N = 1)			H1: No (N = 99)	H1: Yes (N = 1)	
					H2: No	72 (100.00%)	1 (100.00%)	*p* = 1	H2: No	99 (100.00%)	1 (100.00%)	*p* = 1
					H2: Yes	0 (0.00%)	0 (0.00%)		H2: Yes	0 (0.00%)	0 (0.00%)	
Retained products of conception	Not applicable ^a^					H1: No (N = 72)	H1: Yes (N = 1)			H1: No (N = 99)	H1: Yes (N = 1)	
					H2: No	72 (100.00%)	1 (100.00%)	*p* = 1	H2: No	99 (100.00%)	1 (100.00%)	*p* = 1
					H2: Yes	0 (0.00%)	0 (0.00%)		H2: Yes	0 (0.00%)	0 (0.00%)	

*p*—McNemar test, * statistically significant (*p* < 0.05), H1 = identified through primary office hysteroscopy, H2 = identified through secondary office hysteroscopy, ^a^ no such diagnosis was found in this subset.

**Table 3:** The comparison of plasma cells density in tissue samples obtained during primary and follow-up office hysteroscopy among women not treated with antibiotics, among those treated with antibiotics, and irrespective of antibiotic therapy in relation to initially detected intrauterine lesions.

Hysteroscopic Diagnosis	Plasma Cell Density [n/1 HPF]	No Antibiotic Therapy			Antibiotic Therapy			Total		
		H1	H2	*p*	H1	H2	*p*	H1	H2	*p*
Polyp + no features of CE + no CSD	mean ± SD	11.36 ± 9.62	5 ± 8.92	*p* = 0.023 *	12.6 ± 7.33	6.2 ± 5.76	*p* = 0.176	11.75 ± 8.74	5.38 ± 7.89	*p* = 0.009 *
	median	8	2		8	5		8	2	
	quartiles	4–17	0.5–4		8–16	2–10		4.75–16.75	0.75–7	
Polyp + features of CE + no CSD	mean ± SD	6.8 ± 3.96	11.2 ± 8.23	*p* = 0.784	15.32 ± 20.21	7.21 ± 13.58	*p* = 0.033 *	13.12 ± 18.04	7.76 ± 12.42	*p* = 0.082
	median	6	12		7	4		6	5	
	quartiles	4–8	5–14		3.5–20	0–6.5		3–13	1–8	
Features of CE + no polyp/CSD	mean ± SD	5.8 ± 4.55	3 ± 2.35	*p* = 0.265	7.08 ± 12.61	3.41 ± 4.24	*p* = 0.018 *	6.93 ± 11.91	3.36 ± 4.03	*p* = 0.009 *
	median	4	2		4	2		4	2	
	quartiles	3–9	1–5		2–6	0–4		2–6	0–4.5	
CSD	mean ± SD	31.25 ± 42.07	4.75 ± 7.63	*p* = 0.375	12.78 ± 8.18	8.11 ± 10.83	*p* = 0.26	18.46 ± 23.79	7.08 ± 9.77	*p* = 0.116
	median	13	1.5		10	3		10	3	
	quartiles	8.75–35.5	0–6.25		5–19	1–10		5–19	1–10	
No intrauterine lesions	mean ± SD	Not applicable ^a^			28 ± 48.16	6 ± 7.24	*p* = 0.4	25.14 ± 44.61	5.71 ± 6.65	*p* = 0.268
	median				10.5	4.5		10	4	
	quartiles				6.25–12.5	1.75–5.75		6.5–12	2.5–5.5	

*p*—Wilcoxon paired test, * statistically significant (*p* < 0.05), H1 = identified through primary office hysteroscopy, H2 = identified through follow-up office hysteroscopy, ^a^ there was only one woman in this group with values of 8 and 4 in H1 and H2, respectively.

**Table 4:** The comparison of plasma cells density in tissue samples obtained during primary and follow-up office hysteroscopy among women not treated with antibiotics, among those treated with antibiotics, and irrespective of antibiotic therapy in relation to the pattern of abnormal uterine bleeding.

Symptoms	Plasma Cell Density [n/1 HPF]	No Antibiotic Therapy			Antibiotic Therapy			Total		
		H1	H2	*p*	H1	H2	*p*	H1	H2	*p*
No abnormal uterine bleeding	mean ± SD	10.38 ± 8.91	4.12 ± 4.39	*p* = 0.107	12.15 ± 22.64	4.14 ± 5.33	*p* = 0.006 *	11.85 ± 20.88	4.14 ± 5.12	*p* < 0.001 *
	median	7	3		5	2		5	2	
	quartiles	4–12.75	1.75–5		3–10	0–6		3–10	0–5.25	
Heavy menstrual bleeding	mean ± SD	8.09 ± 7.58	8.45 ± 10.33	*p* = 1	14.16 ± 20.1	6.53 ± 13.7	*p* = 0.012 *	11.93 ± 16.72	7.23 ± 12.42	*p* = 0.033 *
	median	5	2		6	1		6	2	
	quartiles	3–9.5	1–13.5		4–15	0–6		3.25–10.75	1–9.25	
Intermenstrual bleeding	mean ± SD	8.67 ± 5.13	5 ± 6.24	*p* = 0.25	5.9 ± 3.51	6.1 ± 3.9	*p* = 1	6.29 ± 3.85	5.5 ± 4.29	*p* = 0.325
	median	10	3		5	5		5	4	
	quartiles	6.5–11.5	1.5–7.5		5–6.5	3.25–8		3.5–8.5	2.25–8	
Heavy and intermenstrual bleeding	mean ± SD	30.75 ± 42.42	2 ± 2.83	*p* = 0.125	13.71 ± 10	8.43 ± 11.75	*p* = 0.297	19.91 ± 25.96	6.09 ± 9.78	*p* = 0.056
	median	12	1		16	5		16	2	
	quartiles	7.25–35.5	0–3		5–22	2–8		6–22	1–6	
Regardless of abnormal uterine bleeding	mean ± SD	12.35 ± 18.12	5.73 ± 7.65	*p* = 0.031 *	11.83 ± 19.35	5.38 ± 8.75	*p* < 0.001 *	11.87 ± 18.88	5.43 ± 8.41	*p* < 0.001 *
	median	8	2		5	3		5	2.5	
	quartiles	4–12.75	1–6		3–10.25	0–7		3–11	0.75–6.25	

*p*—Wilcoxon paired test, * statistically significant (*p* < 0.05), H1 = identified through primary office hysteroscopy, H2 = identified through follow-up office hysteroscopy.

**Table 5:** The persistence of specific histopathological features in the endometrium in primary and follow-up office hysteroscopy among women not treated with antibiotics, among those treated with antibiotics, and regardless of antibiotic therapy.

Histopathological Feature	No Antibiotic Therapy				Antibiotic Therapy				Total			
Lymphocytic infiltration		H1: No (N = 17)	H1: Yes (N = 9)			H1: No (N = 49)	H1: Yes (N = 27)			H1: No (N = 66)	H1: Yes (N = 37)	
	H2: No	15 (88.24%)	6 (66.67%)	*p* = 0.289	H2: No	32 (65.31%)	19 (70.37%)	*p* = 0.868	H2: No	47 (71.21%)	26 (70.27%)	*p* = 0.371
	H2: Yes	2 (11.76%)	3 (33.33%)		H2: Yes	17 (34.69%)	8 (29.63%)		H2: Yes	19 (28.79%)	11 (29.73%)	
Macrophages		H1: No (N = 22)	H1: Yes (N = 4)			H1: No (N = 56)	H1: Yes (N = 20)			H1: No (N = 79)	H1: Yes (N = 24)	
	H2: No	19 (86.36%)	3 (75.00%)	*p* = 1	H2: No	37 (66.07%)	17 (85.00%)	*p* = 0.868	H2: No	57 (72.15%)	20 (83.33%)	*p* = 0.877
	H2: Yes	3 (13.64%)	1 (25.00%)		H2: Yes	19 (33.93%)	3 (15.00%)		H2: Yes	22 (27.85%)	4 (16.67%)	
Granulocytes		H1: No (N = 24)	H1: Yes (N = 2)			H1: No (N = 75)	H1: Yes (N = 1)			H1: No (N = 100)	H1: Yes (N = 3)	
	H2: No	24 (100.00%)	2 (100.00%)	*p* = 0.48	H2: No	74 (98.67%)	1 (100.00%)	*p* = 1	H2: No	99 (99.00%)	3 (100.00%)	*p* = 0.617
	H2: Yes	0 (0.00%)	0 (0.00%)		H2: Yes	1 (1.33%)	0 (0.00%)		H2: Yes	1 (1.00%)	0 (0.00%)	
Spindled transformation and/ or elongation of stromal cells and edema		H1: No (N = 21)	H1: Yes (N = 5)			H1: No (N = 59)	H1: Yes (N = 17)			H1: No (N = 81)	H1: Yes (N = 22)	
	H2: No	15 (71.43%)	4 (80.00%)	*p* = 0.752	H2: No	41 (69.49%)	14 (82.35%)	*p* = 0.596	H2: No	57 (70.37%)	18 (81.82%)	*p* = 0.44
	H2: Yes	6 (28.57%)	1 (20.00%)		H2: Yes	18 (30.51%)	3 (17.65%)		H2: Yes	24 (29.63%)	4 (18.18%)	
Stromal edema and stromal cell compaction		H1: No (N = 25)	H1: Yes (N = 1)			H1: No (N = 59)	H1: Yes (N = 17)			H1: No (N = 84)	H1: Yes (N = 19)	
	H2: No	24 (96.00%)	1 (100.00%)	*p* = 1	H2: No	55 (93.22%)	16 (94.12%)	*p* = 0.014 *	H2: No	79 (94.05%)	18 (94.74%)	*p* = 0.012 *
	H2: Yes	1 (4.00%)	0 (0.00%)		H2: Yes	4 (6.78%)	1 (5.88%)		H2: Yes	5 (5.95%)	1 (5.26%)	
Edema only		H1: No (N = 23)	H1: Yes (N = 3)			H1: No (N = 67)	H1: Yes (N = 9)			H1: No (N = 91)	H1: Yes (N = 12)	
	H2: No	22 (95.65%)	3 (100.00%)	*p* = 0.617	H2: No	48 (71.64%)	6 (66.67%)	*p* = 0.016 *	H2: No	70 (76.92%)	9 (75.00%)	*p* = 0.045 *
	H2: Yes	1 (4.35%)	0 (0.00%)		H2: Yes	19 (28.36%)	3 (33.33%)		H2: Yes	21 (23.08%)	3 (25.00%)	

*p*—McNemar test, * statistically significant (*p* < 0.05), H1 = identified through primary office hysteroscopy, H2 = identified through follow-up office hysteroscopy.

**Table 6:** The analysis of the associations between clinical symptoms, ultrasound findings, and morphological lesions identified during the primary hysteroscopy, and plasma cell density.

Variable	Group	Plasma Cell Density [n/1 HPF]							*p*
		Mean	SD	Median	Min	Max	Q1	Q3	
Abnormal uterine bleeding	No (N = 47)	11.85	20.88	5.0	2	126	3.00	10.00	*p* = 0.561
	Yes (N = 56)	11.89	17.21	6.0	1	94	3.00	13.25	
Heavy menstrual bleeding	No (N = 62)	10.42	18.41	5.0	1	126	3.00	10.00	*p* = 0.153
	Yes (N = 41)	14.07	19.59	8.0	1	94	4.00	19.00	
Intermenstrual bleeding	No (N = 78)	11.74	19.17	5.0	1	126	3.00	10.00	*p* = 0.465
	Yes (N = 25)	12.28	18.34	7.0	1	94	5.00	14.00	
Dysmenorrhea	No (N = 80)	11.39	18.29	5.5	1	126	3.00	11.00	*p* = 0.965
	Yes (N = 23)	13.57	21.17	5.0	1	85	3.50	13.00	
Pelvic pain syndrome	No (N = 101)	12.01	19.04	5.0	1	126	3.00	11.00	*p* = 0.573
	Yes (N = 2)	5.00	2.83	5.0	3	7	4.00	6.00	
Infertility	No (N = 29)	10.90	17.14	5.0	1	94	4.00	10.00	*p* = 0.83
	Primary (N = 27)	9.81	9.31	7.0	1	43	4.00	12.50	
	Secondary (N = 47)	13.66	23.59	5.0	1	126	3.00	10.50	
US Polyp	No (N = 56)	12.16	21.80	5.0	1	126	3.00	10.00	*p* = 0.13
	Yes (N = 47)	11.53	14.90	7.0	1	94	4.00	14.50	
US Polypoid endometrium	No (N = 94)	12.30	19.59	6.0	1	126	3.00	11.00	*p* = 0.392
	Yes (N = 9)	7.44	7.63	4.0	1	25	3.00	10.00	
US Submucosal myoma	No (N = 101)	11.97	19.05	5.0	1	126	3.00	11.00	*p* = 0.952
	Yes (N = 2)	7.00	4.24	7.0	4	10	5.50	8.50	
US Cesarean scar defect	No (N = 92)	11.01	17.90	5.0	1	126	3.00	10.00	*p* = 0.019 *
	Yes (N = 11)	19.09	25.62	10.0	4	94	7.00	17.50	
H1 Polyp	No (N = 56)	9.91	19.18	5.0	1	126	3.00	9.00	*p* = 0.009 *
	Yes (N = 47)	14.21	18.45	8.0	2	94	4.00	17.50	
H1 Polypoid endometrium	No (N = 63)	12.48	20.23	6.0	1	126	4.00	12.00	*p* = 0.437
	Yes (N = 40)	10.93	16.75	5.0	1	94	3.00	10.25	
H1 Micropolyps	No (N = 81)	13.06	20.60	6.0	1	126	4.00	13.00	*p* = 0.095
	Yes (N = 22)	7.50	9.45	5.0	1	43	3.00	7.00	
H1 Endometrial edema	No (N = 102)	11.97	18.95	5.5	1	126	3.00	11.00	*p* = 0.151
	Yes (N = 1)	2.00		2.0	2	2	2.00	2.00	
H1 Focal hyperemia	No (N = 72)	13.10	20.66	6.5	1	126	3.75	13.50	*p* = 0.186
	Yes (N = 31)	9.03	13.80	5.0	1	70	3.00	8.50	
H1 Diffuse hyperemia	No (N = 83)	12.77	20.65	6.0	1	126	4.00	11.50	*p* = 0.417
	Yes (N = 20)	8.15	7.44	4.5	2	25	3.00	10.25	
H1 Retained products of conception	No (N = 102)	11.98	18.94	5.5	1	126	3.00	11.00	*p* = 0.095
	Yes (N = 1)	1.00		1.0	1	1	1.00	1.00	
H1 Intrauterine adhesions	No (N = 98)	12.30	19.26	6.0	1	126	3.25	11.75	*p* = 0.078
	Yes (N = 5)	3.60	1.95	3.0	1	6	3.00	5.00	
H1 Cesarean scar defect	No (N = 90)	10.92	18.03	5.0	1	126	3.00	10.00	*p* = 0.011 *
	Yes (N = 13)	18.46	23.79	10.0	4	94	5.00	19.00	
H1 Submucosal adenomyosis	No (N = 96)	11.38	17.92	5.0	1	126	3.00	11.00	*p* = 0.969
	Yes (N = 7)	18.71	30.29	6.0	2	85	3.00	16.00	
H1 Endometrial elevation	No (N = 97)	12.29	19.37	6.0	1	126	3.00	12.00	*p* = 0.42
	Yes (N = 6)	5.17	2.93	5.0	1	10	4.25	5.75	

*p*—2 groups comparison: Mann–Whitney test; >2 groups comparison: Kruskal–Wallis test + post hoc analysis (Dunn test). * statistically significant (*p* < 0.05), US = identified through ultrasound, H1 = identified through primary office hysteroscopy.

**Table 7:** The analysis of the associations between plasma cell density and the presence of other immune cells in the endometrial infiltrate, as well as the histopathological characteristics of the endometrium obtained during the primary hysteroscopy.

Histopathological Feature	Group	Plasma Cell Density [n/1 HPF]							*p*
		Mean	SD	Median	Min	Max	Q1	Q3	
H1 Lymphocytic infiltration	No (N = 66)	10.41	14.77	5.0	2	94	3.00	10.00	*p* = 0.469
	Yes (N = 37)	14.49	24.61	6.0	1	126	4.00	12.00	
H1 Macrophages	No (N = 79)	11.70	18.92	5.0	2	126	3.00	10.50	*p* = 0.86
	Yes (N = 24)	12.46	19.16	6.0	1	85	3.75	11.25	
H1 Granulocytes	No (N = 100)	11.99	19.10	5.5	1	126	3.00	11.00	*p* = 0.522
	Yes (N = 3)	8.00	9.64	4.0	1	19	2.50	11.50	
H1 Spindled transformation and/or elongation of stromal cells with edema	No (N = 81)	8.70	9.84	5.0	1	70	3.00	10.00	*p* = 0.022 *
	Yes (N = 22)	23.55	34.37	8.0	1	126	5.25	17.25	
H1 Stromal edema and stromal cell compaction	No (N = 84)	13.18	20.57	6.0	1	126	4.00	12.00	*p* = 0.069
	Yes (N = 19)	6.11	5.09	4.0	2	20	3.00	7.50	
H1 Edema only	No (N = 91)	12.35	19.85	6.0	1	126	3.00	11.00	*p* = 0.628
	Yes (N = 12)	8.25	8.02	4.5	2	25	3.75	8.25	

*p*—Mann–Whitney test, * statistically significant (*p* < 0.05), H1 = identified through primary office hysteroscopy.

**Table 8:** The comparison of post-hysteroscopy pregnancy rates and frequencies of IVF utilization between women who received antibiotic treatment and those who did not, both in the entire population of women with infertility and across subpopulations with primary and secondary infertility.

	Parameter		Antibiotic Use			*p*
			No (N = 9)	Yes (N = 65)	Total (N = 74)	
**Any type of infertility**	Post-hysteroscopy pregnancy	No pregnancy	3 (33.33%)	37 (56.92%)	40 (54.05%)	*p* = 0.204
		Live birth	5 (55.56%)	25 (38.46%)	30 (40.54%)	
		Spontaneous miscarriage	1 (11.11%)	3 (4.62%)	4 (5.41%)	
	Post-hysteroscopy pregnancy	No pregnancy	3 (33.33%)	37 (56.92%)	40 (54.05%)	*p* = 0.286
		Pregnancy	6 (66.67%)	28 (43.08%)	34 (45.95%)	
	IVF	No	7 (77.78%)	39 (60.00%)	46 (62.16%)	*p* = 0.468
		Yes	2 (22.22%)	26 (40.00%)	28 (37.84%)	
			**No (N = 5)**	**Yes (N = 22)**	**Total (N = 27)**	
**Primary infertility**	Post-hysteroscopy pregnancy	No pregnancy	1 (20.00%)	14 (63.64%)	15 (55.56%)	*p* = 0.057
		Live birth	3 (60.00%)	8 (36.36%)	11 (40.74%)	
		Spontaneous miscarriage	1 (20.00%)	0 (0.00%)	1 (3.70%)	
	Post-hysteroscopy pregnancy	No pregnancy	1 (20.00%)	14 (63.64%)	15 (55.56%)	*p* = 0.139
		Pregnancy	4 (80.00%)	8 (36.36%)	12 (44.44%)	
	IVF	No	5 (100.00%)	14 (63.64%)	19 (70.37%)	*p* = 0.28
		Yes	0 (0.00%)	8 (36.36%)	8 (29.63%)	
			**No (N = 4)**	**Yes (N = 43)**	**Total (N = 47)**	
**Secondary infertility**	Post-hysteroscopy pregnancy	No pregnancy	2 (50.00%)	23 (53.49%)	25 (53.19%)	*p* = 1
		Live birth	2 (50.00%)	17 (39.53%)	19 (40.43%)	
		Spontaneous miscarriage	0 (0.00%)	3 (6.98%)	3 (6.38%)	
	Post-hysteroscopy pregnancy	No pregnancy	2 (50.00%)	23 (53.49%)	25 (53.19%)	*p* = 1
		Pregnancy	2 (50.00%)	20 (46.51%)	22 (46.81%)	
	IVF	No	2 (50.00%)	25 (58.14%)	27 (57.45%)	*p* = 1
		Yes	2 (50.00%)	18 (41.86%)	20 (42.55%)	
			**No (N = 2)**	**Yes (N = 26)**	**Total (N = 28)**	
**IVF use**	Post-hysteroscopy pregnancy	No pregnancy	0 (0.00%)	15 (57.69%)	15 (53.57%)	*p* = 0.206
		Pregnancy	2 (100.00%)	11 (42.31%)	13 (46.43%)	
			**No (N = 24)**	**Yes (N = 50)**	**Total (N = 74)**	
**No IVF use**	Post-hysteroscopy pregnancy	No pregnancy	17 (70.83%)	31 (62.00%)	48 (64.86%)	*p* = 0.628
		Pregnancy	7 (29.17%)	19 (38.00%)	26 (35.14%)	
			**No (N = 18)**	**Yes (N = 37)**	**Total (N = 55)**	
**Post-hysteroscopy** **symptoms**	Abnormal uterine bleeding	No	14 (77.78%)	32 (86.49%)	46 (83.64%)	*p* = 0.454
		Yes	4 (22.22%)	5 (13.51%)	9 (16.36%)	

*p*—chi-squared or Fisher’s exact test, IVF = in vitro fertilization.

## Data Availability

The database collected during the research has been made publicly available in Harvard Dataverse at https://doi.org/10.7910/DVN/B0BGKU, accessed on 15 April 2025.

## Abbreviations

The following abbreviations are used in this manuscript:

CE	chronic endometritis
RPL	recurrent pregnancy loss
RIF	recurrent implantation failure
OH	outpatient hysteroscopy
AUB	abnormal uterine bleeding
PCD	plasma cell density
CSD	cesarean scar defect
RT-PCR	real-time polymerase chain reaction
RPOCs	retained products of conception
HPF	high-power field
AUC	area under curve
ROC	receiver operating characteristic curve
